# Risk Factors for Sensorineural Hearing Loss and Auditory Maturation in Children Admitted to Neonatal Intensive Care Units: Who Recovered?

**DOI:** 10.3390/children9091375

**Published:** 2022-09-12

**Authors:** Pietro Salvago, Angelo Immordino, Fulvio Plescia, Marianna Mucia, Andrea Albera, Francesco Martines

**Affiliations:** 1 Dipartimento di Biomedicina, Neuroscienze e Diagnostica Avanzata (BiND), Sezione di Audiologia, Università degli Studi di Palermo, Via del Vespro 129, 90127 Palermo, Italy; 2 Dipartimento di Biomedicina, Neuroscienze e Diagnostica Avanzata (BiND), Sezione di Otroinolaringoiatria, Università degli Studi di Palermo, Via del Vespro 129, 90127 Palermo, Italy; 3 Dipartimento di Promozione della Salute, Materno-Infantile, di Medicina Interna e Specialistica di Eccellenza “G. D’Alessandro”, University of Palermo, Piazza delle Cliniche 2, 90127 Palermo, Italy; 4 UOSD Audiologia, A.O.U.P. “Paolo Giaccone”, Via del Vespro 129, 90127 Palermo, Italy; 5 Dipartimento di Scienze Chirurgiche, Sezione di Otorinolaringoiatria, Università degli Studi di Torino, Corso Dogliotti 14, 10126 Torino, Italy

**Keywords:** NICU, sensorineural hearing loss, auditory maturation

## Abstract

Background: Newborns admitted to neonatal intensive care units (NICUs) are at higher risk of developing sensorineural hearing loss (SNHL), which may improve over time. The aim of this study was to describe the prevalence of the main risk factors for SNHL in a NICU cohort, focusing on children who underwent auditory maturation. Methods: An observational study of 378 children admitted to NICUs, who were followed for at least 18 months, with periodic audiologic assessments. Results: Out of 378 patients, 338 had normal hearing and 40 were hearing-impaired; we found a higher percentage of extremely preterm (EPT) and extremely low-birthweight (ELBW) infants in SNHL children (*p* < 0.05). Seventeen infants presented auditory improvement, with a mean maturation time of 6.17 months. A significant difference emerged between patients with stable SNHL and those who improved only in the case of hyperbilirubinemia (*p* = 0.005). The initial hearing threshold was a predictor of auditory improvement and moderately correlated to the time of auditory maturation (*p* = 0.02). Conclusions: Our study supports the trend toward recognizing worse prognoses and slower maturation processes among NICU children who suffer from severe to profound SNHL. Caution must be taken when deciding on earlier cochlear implantation.

## 1. Introduction

It has been estimated that permanent bilateral sensorineural hearing loss (SNHL) involves 1–3/1000 live births in wellborn infants, increasing up to 50-fold in children who are at risk [1,2]; in particular, 2–4/100 infants who are admitted to neonatal intensive care units (NICUs) may develop hearing impairment [3,4].

Many perinatal problems may require NICU admission, including hyperbilirubinemia, ototoxic drug use, TORCH infections, meningitis, mechanical ventilation, and periventricular hemorrhage, making the immature brain more sensitive to damage [5]. In addition, prematurity and low birthweight can affect hearing pathway maturation and the ability to discriminate among sounds which are critical to speech, language, auditory processing, and reading development [6]. The more premature a baby is born, the greater these risks. It has been previously demonstrated that the risk for SNHL is highest for very premature babies, born before 32 weeks of pregnancy and having a birthweight of less than 1500 g [7,8].

The maturation of the auditory system is characterized by two different stages: the first lasts until the sixth month of gestation and involves the complete development of the peripheral part of the auditory system; the second includes synaptogenesis and the continued refinement of the central part of the auditory system, which continues until the second year of life [9,10]. The multiple risk factors which are associated with babies who survive their stay in the NICU may injure the auditory pathway by disrupting transmission from the cochlea to the central parts of the auditory system [8].

In the first year of life, the hearing threshold is not always stable [3,11]. Modifications in the hearing threshold of NICU children over time (including worsening, improvement, or complete recovery) require long-term hearing follow-up and early intervention, especially for patients who are candidates for cochlear implantation [12,13]. Hearing may change because of either resolved middle-ear effusion or recovery from minor central neurological disorders. However, this improvement may also be due to a delayed maturation of the immature central auditory pathways, a phenomenon frequently observed in very preterm neonates [14,15,16,17]. Reversible abnormalities are also recognized in healthy infants, and the absence of risk factors and diseases in such cases makes it harder to explain the pathological mechanisms behind their impaired auditory maturation [12].

Early rehabilitative treatment is essential to providing adequate access to the sound environment; however, prompt audiological diagnoses are not always reliable [12,18]. Even if a child with congenital severe-to-profound SNHL receives hearing aids as soon as possible, followed by cochlear implant within the first year of life [19], it is still a matter of debate whether to further wait to recognize signs of auditory maturation that may contraindicate surgical intervention.

Previous investigations have attempted to identify predictive factors for auditory maturation among at-risk infants, though with contradictory results. Hof et al. (2013) hypothesized that hearing improvement in cases of initially identified SNHL is more frequent among infants with a gestational age of <28 weeks [13], while Yang et al. found auditory improvement also in children who were born after 29 weeks’ gestational age [17]. In addition, Frezza et al. observed worse hearing prognoses in extremely preterm (EPT) children, especially when they had experienced prolonged NICU stays [20].

The purpose of our investigtion was to describe the prevalence of the main risk factors for SNHL in a NICU cohort and to understand whether there are differences in their distribution between normal-hearing babies and infants with stable and unstable hearing loss; in addition, we focused on identifying the main characteristics of children who experienced hearing threshold improvement.

## 2. Materials and Methods

### 2.1. Participants

From January 2017 to February 2021, 436 children who were admitted to the NICU for more than 5 days were enrolled at the Department of Audiology of the Policlinico “P. Giaccone” University Hospital of Palermo, Italy. All patients included were referred to our service for audiological evaluation because of the presence of risk factors for SNHL according to the American Joint Committee on Infant Hearing (JCIH) guidelines [21].

Out of these 436 patients, 394 (90.36%) participated in this study from the start, but we lost touch with 16 infants during follow-up monitoring. The final response rate was 86.69%, corresponding to 378 infants. The mean age at the time of the first appointment was 4 weeks’ corrected age (CA).

After approval by the Hospital Ethics Committee (Approval number: 12/16), the study protocol was fully explained to the children’s parents and informed consent was obtained for each patient.

Parents of the children were informed about the importance of their children’s participation in the audiological follow-up and were invited to complete a questionnaire administered by a trained staff member to assess the following risk factors for SNHL: prematurity (gestational age ≤ 37 weeks), very low birthweight (<1500 g, VLBW), respiratory distress (e.g., perinatal asphyxia, mechanical ventilation > 5 days, extracorporeal membrane oxygenation, infant respiratory distress syndrome), hyperbilirubinemia (serum bilirubin > 10 mg/dL), pregnant maternal infection (TORCH), perinatal infections (e.g., sepsis and meningitis), and exposure to ototoxic drugs (furosemide, dexamethason, vancomycin, gentamycin, and tobramycin). Exclusion criteria were: family history of hearing impairment, GJB2 gene mutation, neurological disorders (e.g., periventricular leukomalacia, intraventricular hemorrhage, hydrocephalus, cerebral palsy, encephalopathy), cranio-facial abnormalities, and syndromes associated with hearing impairment or permanent conductive hearing loss.

### 2.2. Procedure

An experienced audiologist and otorhinolaryngologist examined the condition of the external auditory canal and tympanic membrane with otoscopy, along with the nose, throat, head, and face to rule out any ear anomalies or syndromic features related to hearing impairment.

The same qualified biomedical staff member assessed all children using auditory brainstem responses (ABR), transient-evoked otoacoustic emissions (TEOAEs), and tympanometry. In addition, depending on the child’s age and whether the child could be conditioned, patients were evaluated with either behavioral observation audiometry (BOA) or visual reinforcement audiometry (VRA).

ABR recording was performed through an AMPLAID mk22 auditory-evoked potentials device in a soundproof room; all children were in natural sleep or in calm conditions throughout the evaluation. After adequate preparation of the skin, silver recording electrodes were attached to the upper forehead (recording electrode), the ipsilateral mastoid process (reference electrode), and contralateral mastoid process (ground electrode). Thus, the Fpz–M1–M2 electrode montage was used for recording ABR. Unfiltered full square-wave pulses of 100 ms duration were used, with alternating polarity. The clicks were delivered monaurally by a handheld TDH-49 headphone at a rate of 21/s. The analysis time was 15 ms. The recording bandwidth for click threshold determination was 100–2500 Hz. Electrode and interelectrode impedance were ensured to be below 5 kHz and 2 kHz, respectively. Each run consisted of summing the responses to 2000 clicks. Click stimuli were presented starting at a level of 100 dB HL. With step sizes of 10 dB, the level was decreased until no response was found. The response threshold was estimated by the lowest level at which a response was recorded. An infant was considered to have passed the ABR test if a replicable wave V response was recordable at 30 dB HL in both ears, whereas SNHL was defined as elevated ABR response thresholds (≥40 dB) in one or both ears. ABR thresholds at 20 dB HL or lower were considered normal, thresholds between 30 db HL and 40 dB HL were considered as indicative of mild hearing loss, thresholds between 50 db HL and 60 dB HL as moderate hearing loss, thresholds between 70 db HL and 80 dB HL as severe hearing loss, and thresholds higher than 90 dB HL as profound hearing loss [22].

Experienced clinical audiologists interpreted the ABR response waves. The response latencies (in milliseconds) were obtained by establishing the peak of the wave and reading out the digitally displayed time.

TEOAEs were recorded using the Otodynamics ILO 288 USB II device with standard settings; the stimulus was a nonlinear click at an intensity of 84 dB SPL, and a number of 260 averages was used. TEOAEs were considered ‘‘PASS’ only when the reproducibility of the recorded emissions exceeded 70% in at least three octave bands and the stimulus stability exceeded 80%. A second TEOAEs test was also performed in re-examined infants if the middle ear was free from disease [16].

Tympanometry was performed with an Inventis Flute Diagnostic Middle Ear Analyzer system using the standard settings and a 226/1000 Hz probe tone (depending on the age) and an air pressure range of −400 to +200 daPa with automatic recording.

BOA and VRA were performed with a pediatric audiometer (Interacoustics PA5) at 500, 1000, 2000, and 4000 Hz, with 20 and 80 dB being the minimum and maximum levels assessed by the device. To obtain responses, stimuli were presented in decreasing order in the right and left lateral planes, with or without visual conditioning.

All patients included in the study had a minimum follow-up of 18 months from the time of the first appointment. The frequency of audiological examinations depended on the age and hearing status of the child. Evaluations of patients diagnosed with hearing loss were performed 3–4 times a year for children under 2 years of age and 2–3 times a year for children from 2 to 5 years of age; infants without any hearing impairment were assessed 2–3 times a year during their first year of life, followed by annual examinations as they got older [23].

Each child was recommended to wear hearing aids (HAs) if their ABR thresholds were 40 dB nHL or worse (up to 95 nHL) in their best ear. HAs were provided at a mean age of 3.4 ± 0.6 months. Subjects who did not benefit from hearing aid amplification and who met criteria for cochlear implant (CI) candidacy were referred for CI surgery.

A ‘maturated’ ABR was hypothesized when the following conditions were satisfied: (1) a unilateral or bilateral wave V identifiable over 30 dB nHL during the first clinical ABR; (2) the psychoacoustic threshold improved by 20 dB nHL or more with respect to the previous ABR threshold.

### 2.3. Statistical Analysis

Continuous variables were represented as mean ± standard deviation (SD) and categorical variables as number and percentage. Comparisons between categorical variables were performed using the chi-square test and Fisher’s exact test. The Mann–Whitney U test was used to analyze continuous variables without normal distribution. Spearman’s correlation coefficients were calculated. Simple logistic regression (SLR) analysis was performed, considering hearing threshold amelioration as the dependent variable and prematurity, VLBW, and ABR initial threshold as independent variables. A two-tailed *p* < 0.05 was considered significant.

## 3. Results

Table 1 summarizes the main clinical characteristics of the whole sample studied. Ultimately, 378 patients, 223 males (58.99%) and 155 females (41.01%), were included, with a sex ratio of 1.43 and a mean follow-up of 20.11 ± 1.69 months. Subjects were classified into two groups according to their hearing status: the first, composed of 338 patients (89.41%), presented normal hearing; the second group included 40 individuals (10.59%) affected by SNHL. We identified 155 (45.85%) full-term babies in the first group and 19 (47.5%) in the second group.

We found a higher percentage of EPT and ELBW infants in the SNHL group with respect to normal-hearing children, with a statistically significant difference (*p* < 0.05). Hearing-impaired children also showed a higher prevalence of prenatal (4.49%) and perinatal infections (9.52%) compared to normal-hearing infants (*p* < 0.0001). No significant differences between the groups were found in terms of respiratory distress, hyperbilirubinemia, and ototoxic drug exposure (*p* > 0.05).

Out of 40 SNHL patients, 6 were recognized as unilateral and 34 as bilateral at their initial ABR, with a higher percentage of bilateral SNHL (95.65%) among children with stable hearing loss (Table 2). Seventeen (42.5%) infants presented an improvement in their hearing threshold, with a mean time of maturation of 6.17 ± 4.59 months (Table 3). Eight patients exhibited signs of auditory maturation before six months of follow-up, while nine continued to improve after. Twenty-seven ears presented a better hearing threshold at the end of the follow-up, with fifteen ears (55.55%) recovering completely. With the exception of one patient with profound, stable, single-sided deafness, the other five infants with unilateral SNHL (two moderate, two severe and one profound hearing impairment) experienced hearing threshold change. Only one case of mild bilateral auditory deterioration was found in a female patient.

The study of auditory threshold in the SNHL group evidenced a mean hearing threshold of 74.87 db HL in the right ear and 70.75 db HL in the left ear at the first evaluation, and a mean hearing threshold of 61 db HL in the right ear and 55.25 db HL in the left ear (*p* < 0.05) in the final evaluation. When focusing on patients with maturation of auditory pathways, we recognized an initial mean hearing threshold of 63.82 db HL in the right ear and 62.35 db HL in the left ear, and a final mean hearing threshold of 33.52 db HL in the right ear and 26.47 db HL in the left ear (*p* < 0.001); a mean improvement of more than 30 db HL was evidenced in both ears.

Initial hearing threshold was shown to be weakly correlated to gestational age, with a higher hearing threshold as gestational age decreased (ρ = −0.373, *p* = 0.014).

We found a lower (35.29%) percentage of infants with severe SNHL in the better ear among patients who underwent hearing threshold improvement with respect to those who did not (56.52%), whether or not significant (*p* = 0.21). The analysis of SNHL risk factors evidenced a significant difference only in the case of hyperbilirubinemia between patients with stable hearing loss and individuals who improved (*p* = 0.005), with the former showing a higher prevalence (47.82%) of abnormal bilirubin serum levels. No significant correlation was observed between hearing improvement and time of maturation (ρ = 0.036, *p* = 0.85), while a moderate positive correlation between initial ABR threshold and time of maturation was recognized (ρ = 0.408, *p* = 0.02).

Out of 17 patients who exhibited an improvement in their hearing threshold, 4 cases presented an association between recordable TEOAEs and abnormal ABR, with a suspected pattern of auditory neuropathy spectrum disorder (ANSD); all infants with stable SNHL had absent TEOAEs. In particular, only 10 ears were PASS at the initial TEOAEs screening, while 27 were PASS at the final examination (*p* = 0.001).

Additionally, the most represented risk factor for SNHL among infants with auditory maturation was respiratory distress (76.47%), while the least frequent was hyperbilirubinemia (5.88%). All cases of prenatal infection were caused by cytomegalovirus (CMV) infection.

Simple logistic regression analysis did not show any statistically significant association between prematurity (C.I. 0.89–1.33, *p* = 0.33), VLBW (C.I. 0.98–1.00, *p* = 0.19), and improvement in hearing threshold. However, initial ABR hearing threshold was found to be a predictor of auditory improvement: children who showed worse hearing thresholds were less likely to experience maturation of the auditory pathways (C.I. 0.95–0.99, *p* = 0.02).

## 4. Discussion

Multiple factors are responsible for NICU admission; children who require intensive care may develop different forms of hearing impairment and, because of their brainstem susceptibility, they may experience significant fluctuations in their hearing threshold. Of course, a comprehensive audiological assessment is mandatory to rule out any hearing loss that may affect speech and language development, but cases of unstable hearing function can represent a challenge for clinicians; in fact, auditory maturation involving infants with severe-to profound SNHL can change the rehabilitative indications to the point of avoiding surgical candidacy.

Several studies have previously investigated hearing threshold change among NICU babies but, due to the small percentage of cases of hearing improvement, data are still not conclusive. Factors such as gestational age, birthweight, length of NICU stay, and degree of hearing loss have been identified as possible prognostic factors, though with contradictory results [13,17,20]. Other studies instead focused on ABR electrophysiological findings in newborns who were admitted to NICUs, demonstrating a significant relationship between delayed interpeak latencies, male sex, and prematurity [24,25].

**Figure 1:**
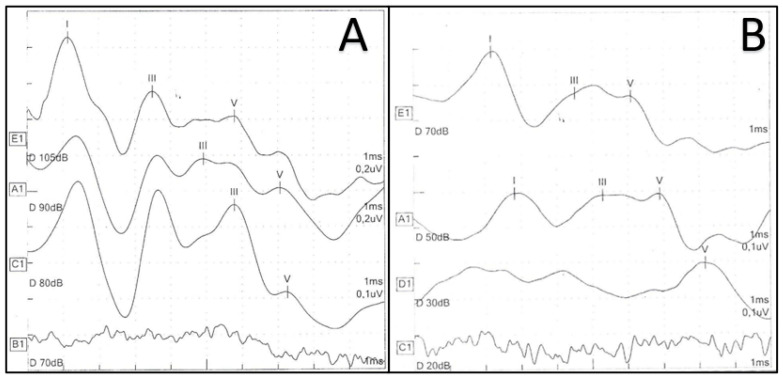
Example of hearing threshold improvement of the right ear from 80 db nHL at the first ABR (**A**) to 30 db nHL at the last ABR (**B**).

Analyzing our data, 4.49% of the cases showed hearing threshold change among NICU infants (Figure 1), which is in line with a previous investigation [20]. Differently from our cohort, Ciorba et al. recognized a lower rate (1.3%) of NICU infants who initially failed the neonatal hearing screening and had normal hearing during the audiological follow-up; however, they hypothesized auditory maturation only in the case of a bilateral wave V identifiable within 30 db nHL until the end of the follow-up and included patients with risk factors of genetic syndromes and congenital malformations who were excluded from our sample [26].

Children who experienced SNHL tended to suffer from a higher degree of prematurity and lower birthweight than normal-hearing infants, as well as a higher rate of pre- and postnatal infections (*p* < 0.05). It may be supposed that the severity of the interacting factors affecting NICU infants might play a crucial role in the development of SNHL. With the exception of hyperbilirubinemia (*p* = 0.005), we did not find any significant difference in the prevalence of risk factors between children with stable SNHL and infants with auditory maturation; due to their immature brain barrier, NICU preterm children are less likely to recover from hearing loss induced by hyperbilirubinemia [27]. However, as reported by Talero-Gutierrez et al., it is difficult to find a clear etiology that may be recognized as the determining factor differentiating children who improved from those who did not. In fact, several possible etiological perinatal and postnatal factors could be claimed as interfering with auditory maturation processes even in the same patient [28].

Interestingly, it seems that those showing worse initial hearing thresholds had a reduced chance of experiencing hearing improvement (*p* = 0.02) and a slower process of maturation of auditory pathways, regardless of when it occurred (*p* = 0.02). These results may be read in light of the numerous mechanisms interfering with auditory plasticity in NICU infants; in fact, different and heterogenous conditions may transitorily or permanently injure a susceptible auditory brainstem, negatively influencing the pathway’s maturation or regeneration [12].

If we restrict our analysis to preterm SNHL children (15 patients), our cohort could be compared to the study by Hof et al., with a similar percentage of respiratory distress syndrome but a lower number of infants exposed to ototoxic drugs. Even if we observed a lower number of cases with hearing improvement (only three among preterm babies), we recognized the same number of complete recoveries from hearing impairment. Differently from these authors, though, no significant relationship between auditory maturation and gestational age was found; we should underline that the results may be influenced by the different timing of initial ABR (half of the children in Hof et al.’s sample were initially assessed before the 42nd week of gestational age), which could have led to the overestimation of the frequency of transitory auditory dysfunction [14].

Similarly to Frezza et al., we observed a significant difference in terms of gestational age and birthweight between normal-hearing and SNHL infants. When comparing data regarding patients with hearing threshold change, it also emerged that, in their study, children with a higher degree of hearing loss needed a longer time to reach a normalization of their hearing threshold; this finding supports the evidence of a positive correlation between ABR threshold and time of improvement (*p* = 0.02). In addition, it was clear that patients with severe-to-profound hearing loss had a worse prognosis, corroborating the possible role of hearing threshold as a predictive factor (*p* = 0.02). This result is further supported by Yang et al., who longitudinally assessed 64 infants who failed the TEOAEs screening test, concluding that patients with a lower initial auditory steady-state response (ASSR) threshold were more likely to show improved hearing over time [17].

In contrast to Frezza et al., an investigation performed by Bovo et al. reported several cases of preterm infants with severe-to-profound hearing loss who showed an amelioration of their hearing threshold [12]; however, various comorbidities and the timing of the initial ABR may partially explain this difference. In addition, it is evident that improvement is a dynamic and unpredictable process that, in the majority of cases, starts within the sixth month of CA and may continue after twelve months of CA.

Four cases of transient ANSD were recognized among children who underwent hearing threshold improvement in our sample. Three of them reached a normalization of their hearing threshold, while one presented a complete recovery of the ANSD ear, with the SNHL ear presenting stable profound hearing impairment. Savenko et al., in their longitudinal cohort study on preterm infants, also reported three cases of moderate ANSD who had normal hearing at the end of the follow-up; differently from our investigation, they identified few children who initially suffered from ANSD but subsequently transformed to SNHL and vice versa [23]. In addition, not every patient who presented an ANSD pattern was preterm because two of them presented other risk factors. All patients showing present TEOAEs at the beginning of our study experienced a normalization of the hearing threshold but, due to the limited number of cases, we were not able to determine whether TEOAE status may play a role as a predictor of hearing improvement.

With the exception of one patient whose initial ABR may suggest a future cochlear implant candidacy, all of the hearing-impaired children who presented an amelioration in hearing were fitted with hearing aids whenever appropriate. In particular, three of them continued to improve until 12 months of follow-up and after.

Our study presents the following limitations. To begin with, the number of cases with maturation of the auditory pathways is small, although it is in line with previously published data. We opted for excluding some SNHL risk factors (e.g., genetic SNHL) because they may increase the number of cases of stable hearing loss due to their rare tendency to transform into a normal hearing threshold. In addition, we did not include the length of NICU stay, thus excluding some intrinsic NICU risk factors (e.g., noise exposure) that may affect our results.

In summary, it is clear that audiological follow-up of NICU children is a challenging diagnostic process in which the clinician needs to promptly recognize any sign of auditory improvement/deterioration in order to implement the best rehabilitative strategy according to the hearing status and age of the patient; apart from the initial hearing threshold, it is difficult to find strong predictors of hearing improvement because children who are admitted to the NICU often present numerous risk factors whose interaction might partially explain the evolution of hearing threshold over time. It should be kept in mind that even cases of profound SNHL can transform into normal hearing, and, for this reason, waiting until 80–85 weeks of gestational age before deciding on cochlear implantation is still a good cut-off time, especially in preterm infants [12,14,29,30].

## 5. Conclusions

Our study supports the trend toward recognizing a worse prognosis and a slower process of maturation among NICU children who suffer from severe-to-profound hearing impairment. Of course, being premature and having a VLBW means being at a higher risk of incomplete maturation of the auditory brainstem; nevertheless, the multiple interacting factors that characterize NICU newborns, acting on a susceptible auditory nervous system, may further influence the chance of auditory improvement. Caution must be taken when deciding on earlier cochlear implantation because auditory maturation after birth is still an unpredictable and dynamic process; future research is needed to better clarify the prognostic factors that may further assist audiologists in the management of NICU hearing-impaired children and better address parental concerns.

## Figures and Tables

**Table 1:** Demographic characteristics and risk factors of the NICU cohort.

Cohort	Normal Hearing		Hearing Impairment		Total		Statistical Analysis
	*n*	(%)	*n*	%	*n*	%	
**Gender**							ns
**Total**	338	-	40	-	378	-	
**Male**	196	(57.9)	27	(67.5)	223	(58.99)	
**Female**	142	(43.1)	13	(32.5)	155	(41.01)	
**Risk Factors**							
**Prematurity**							χ^2^ = 11.06 *p* = 0.004 *
**MLPT (32–37 W)**	98	(28.99)	10	(25)	108	(28.57)	
**VPT (28–31 W)**	57	(16.86)	2	(5)	59	(15.6)	
**EPT ≤ 27**	28	(8.28)	9	(22.5)	37	(9.78)	
**Birthweight**							0.001
**VLBW**	68	(20.11)	3	(7.5)	71	(18.78)	
**ELBW**	20	(5.91)	7	(17.5)	27	(7.14)	
**Respiratory distress**	204	(60.35)	26	(65)	230	(60.84)	ns
**Hyperbilirubinemia**	92	(27.21)	12	(30)	104	(27.51)	ns
**Prenatal infections**	10	(2.95)	7	(17.5)	17	(4.49)	χ^2^ =17.6 *p* < 0.0001
**Perinatal infections**	24	(7.1)	12	(30)	36	(9.52)	χ^2^ = 21.76 *p* < 0.0001
**Ototoxic drugs**	68	(20.11)	13	(32.5)	81	(21.42)	ns

Abbreviations: MLPT: moderate–late preterm; VPT: very preterm; EPT: extremely preterm; VLBW: very low birthweight; ELBW: extremely low birthweight; ns: not significant. * The test refers to the comparison between the three degrees of prematurity.

**Table 2:** Comparative study of children with permanent hearing loss and infants with auditory improvement.

Cohort	Auditory Maturation		Stable Hearing Loss		Statistical Analysis
	*n*	(%)	*n*	(%)	
**Gender**					ns
**Total**	17	(-)	23	-	
**Male**	11	(64.7)	16	(69.56)	
**Female**	6	(35.3)	7	(30.43)	
**Audiological Characteristics**					
**Ear involvement**					ns
**Unilateral**	5	(29.41)	1	(4.35)	
**Bilateral**	12	(70.59)	22	(95.65)	
**Hearing loss degree**					ns *
**Medium/Medium**	2	(11.76)	7	(30.43)	
**Medium/Severe**	4	(23.52)	1	(4.35)	
**Medium/Profound**	-	-	2	(8.69)	
**Severe/Severe**	4	(23.52)	4	(17.39)	
**Severe/Profound**	1	(5.88)	3	(13.04)	
**Profound/Profound**	1	(5.88)	6	(26.08)	
**Risk Factors**					
**Prematurity**					ns **
**MLPT (32–37 W)**	5	(29.41)	5	(21.73)	
**VPT (28–31 W)**	-	-	2	(8.69)	
**EPT ≤ 27**	2	(11.76)	7	(30.43)	
**Birthweight**					ns
**VLBW**	-	-	2	(8.69)	
**ELBW**	2	(11.76)	5	(21.73)	
**Respiratory distress**	13	(76.47)	15	(65.21)	ns
**Hyperbilirubinemia**	1	(5.88)	11	(47.82)	φ = 0.45 *p* = 0.005
**Prenatal infections**	5	(29.41)	2	(8.69)	ns
**Perinatal infections**	6	(35.3)	6	(26.08)	ns
**Ototoxic drugs**	5	(29.41)	8	(34.78)	ns

Abbreviations: MLPT: moderate–late preterm; VPT: very preterm; EPT: extremely preterm; VLBW: very low birthweight; ELBW: extremely low birthweight; ns: not significant. * The test refers to the comparison between the six SNHL pattern. ** The test refers to the comparison between the three degrees of prematurity.

**Table 3:** Audiological findings in patients with observed maturation of auditory pathways.

Patient	Gender	Initial ABR Threshold		Last ABR Threshold		Initial TEOAEs Right/Left	Last TEOAEs Right/Left	Risk Factors	Time of Maturation (Months)
		Right	Left	Right	Left				
1	M	50	70	20	20	REFER/REFER	PASS/PASS	MLPT, respiratory distress, hyperbilirubinemia	5
2	M	70	20	50	20	REFER/PASS	REFER/PASS	Respiratory distress	10
3	M	20	60	20	20	PASS/PASS	PASS/PASS	EPT, ELBW, respiratory distress, hyperbilirubinemia, perinatal infection, ototoxic drugs	2
4	F	70	70	20	20	PASS/PASS	PASS/PASS	CMV	6
5	M	50	50	20	20	PASS/PASS	PASS/PASS	MLPT, CMV	3
6	F	70	20	30	20	REFER/PASS	PASS/PASS	Respiratory distress, CMV, perinatal infection, ototoxic drugs	4
7	F	70	70	30	30	REFER/REFER	PASS/PASS	EPT, ELBW, respiratory distress	2
8	M	20	120	20	20	PASS/REFER	PASS/PASS	Respiratory distress	18
9	M	120	80	120	20	REFER/PASS	REFER/PASS	Respiratory distress, perinatal infection, ototoxic drugs	6
10	M	70	70	50	60	REFER/REFER	REFER/REFER	Respiratory distress	12
11	M	60	50	20	20	REFER/REFER	PASS/PASS	MLPT, respiratory distress, perinatal infection, ototoxic drugs	2
12	F	70	70	40	40	REFER/REFER	REFER/REFER	MLPT	4
13	F	70	60	20	30	REFER/REFER	PASS/PASS	Respiratory distress, perinatal infection, ototoxic drugs	2
14	F	105	90	30	40	REFER/REFER	PASS/REFER	MLPT, Respiratory distress, perinatal infection	6
15	M	70	50	30	20	REFER/REFER	PASS/PASS	Respiratory distress	9
16	M	30	60	30	30	REFER/REFER	PASS/PASS	Respiratory distress, CMV	12
17	M	70	50	20	20	REFER/REFER	PASS/PASS	CMV	2

Abbreviations: MLPT: moderate–late preterm; EPT: extremely preterm; ELBW: extreme low birthweight; CMV: cytomegalovirus.

## Data Availability

Not applicable.
